# SIRT2 inhibition enhances mitochondrial apoptosis in *Brucella*-infected bovine placental trophoblast cells

**DOI:** 10.1186/s13567-025-01518-8

**Published:** 2025-05-02

**Authors:** Mengyu Zhang, Lin Qi, Junmei Li, NingQiu Yuan, Yunyi Zhai, Mingyue Hao, Dong Zhou, Wei Liu, Yaping Jin, Aihua Wang

**Affiliations:** 1 College of Veterinary Medicine, Northwest A&F University, Yangling District, Xianyang, 712100 China; 2 Key Laboratory of Animal Biotechnology of the Ministry of Agriculture, Northwest A&F University, Yangling District, Xianyang, 712100 China

**Keywords:** *Brucella*, bovine placental trophoblast cells, SIRT2, mitochondrial apoptosis

## Abstract

*Brucella* is a successful pathogen that employs a plethora of immune evasion mechanisms. This contributes to pathogenesis and persistence and limits the efficacy of available treatments. An increasing understanding of host‒pathogen interactions suggests that integrating host-directed strategies with existing anti-*Brucella* treatments could lead to more effective bacterial clearance and a reduction in drug-resistant strains. SIRT2 is a nicotinamide adenine dinucleotide (NAD^+^)-dependent deacetylase found in mammals. It can deacetylate various transcription factors and regulatory proteins, playing crucial roles in host‒pathogen interactions and pathogen infection-induced apoptosis. In this study, we investigated the role of SIRT2 in *Brucella*-induced cell apoptosis using bovine placental trophoblast cells. Our results indicate that *B. abortus* A19 infection upregulates SIRT2 protein expression and significantly induces mitochondrial apoptosis in these cells. Furthermore, inhibition of SIRT2 exacerbates *B. abortus* A19-induced mitochondrial apoptosis and markedly inhibits intracellular bacterial survival. These results prove the role of SIRT2 in *Brucella* pathogenesis and the mechanism of action.

**Supplementary Information:**

The online version contains supplementary material available at 10.1186/s13567-025-01518-8.

## Introduction

*Brucella* spp. are Gram-negative, facultative intracellular bacteria belonging to the class *Alphaproteobacteria*. Brucellosis, caused by *Brucella* spp., is a major zoonotic disease worldwide. In animals, it can lead to abortion and male infertility, whereas in humans, it presents with diverse symptoms, including Malta fever, night sweats, anorexia, polyarthritis, meningitis, and pneumonia [1]. Currently, there is no internationally recognized safe and effective brucellosis vaccine in humans [2]. Traditional treatment of brucellosis involves the use of multiple antibiotics, leading to an increase in drug-resistant cases [3]. This indicates a significant limitation of relying solely on microbial-targeted therapy.

*Brucella* can invade various host cells, including macrophages and trophoblasts [4]. To survive and proliferate in harsh intracellular environments, *Brucella* has evolved various virulence factors or systems to evade immune response mechanisms, such as lipopolysaccharides (LPS), the type IV secretion system (T4SS), and outer membrane proteins (OMPs) [5–7]. The immune system consists of innate immunity and adaptive immunity [8, 9]. Innate immunity serves as the first line of defence against pathogen invasion and relies on the host’s pattern recognition receptors (PRRs) to identify pathogen-associated molecular patterns (PAMPs). This recognition triggers the production of interferons (IFNs) and inflammatory cytokines to eliminate pathogens [10–13]. As the second line of defense, adaptive immunity enhances antibody production by activating antigen-presenting cells such as dendritic cells, phagocytes, and cytotoxic lymphocytes. During long-term interactions with the host, *Brucella* species have evolved stealth strategies to evade, disrupt, or suppress immune responses, ultimately leading to chronic infections.

SIRT2 is a mammalian nicotinamide adenine dinucleotide (NAD^+^)-dependent deacetylase that is primarily localized in the cytoplasm and functions as the only major cytoplasmic sirtuin protein [14]. Its activities are largely attributed to deacetylation targets within the cytoplasm. However, during mitosis and pathogen infection, SIRT2 shuttles to the nucleus and regulates chromatin condensation [15]. Recent research has revealed that a small amount of SIRT2 is localized to mitochondria, where it participates in the regulation of mitochondrial fission and fusion [16]. SIRT2 interacts with numerous substrates, including histones and nonhistone proteins, to regulate their biological activities. Its biological functions involve mainly the regulation of host‒pathogen interactions, the cell cycle, metabolism, cancer and immunity [17–22]. The antibacterial effects of the SIRT2 inhibitor AGK2 have been confirmed in *Mycobacterium tuberculosis*, *Listeria*, *Staphylococcus aureus*, and *Salmonella* [17, 23–26]. In infections caused by *Mycobacterium tuberculosis* and *Listeria*, SIRT2 activity promotes bacterial survival and infection [17, 23]. In *Staphylococcus aureus* infection, SIRT2 may exacerbate the severity of the infection [26]. For *Salmonella* infection, SIRT2 plays a dual role, assisting in bacterial clearance while potentially weakening host resistance [27]. However, the role of SIRT2 in *Brucella* infection remains unclear, and further research is needed to elucidate its role in *Brucella* infection.

In this study, we used multiple methods to demonstrate that *B. abortus* A19 infection leads to the upregulation of SIRT2 protein expression in bovine placental trophoblast cells, which significantly induces apoptosis and mitochondrial damage. Inhibition of SIRT2 exacerbated *B. abortus* A19-induced mitochondrial apoptosis, significantly inhibiting bacterial intracellular survival. These findings elucidate the biological function of SIRT2 in *Brucella* infection and provide a theoretical basis for exploring effective host-directed therapies for brucellosis.

## Materials and methods

### Biosafety statement

All experiments were conducted in accordance with the “Regulations on Biosafety of Pathogenic Microorganism Laboratory” (2004) No. 424 issued by the State Council of the People’s Republic of China and were approved by the Northwest A&F University Biosafety Committee.

### Bacterial strains and mammalian cells

The *B. abortus* vaccine strain A19 was provided by the Shaanxi Veterinary Drug Supervision Institute and cultured on tryptic soy agar (TSA) for 72 h or in tryptic soy broth (TSB) with shaking for 48 h. All procedures involving live *B. abortus* A19 strains were conducted in a biosafety level 3 (BSL-3) facility. Bovine placental trophoblast cells (BTCs) were obtained from Beijing Agricultural College and cultured in DMEM/F-12 medium containing 10% fetal bovine serum (Thermo Scientific, Waltham, MA, USA) at 37 ℃ with 5% CO_2_.

### BTC infection assay and AGK2 treatment

BTCs in the logarithmic growth phase were seeded into 6-well plates at a density of 4 × 10^5^ cells per well and cultured for 12 h. *B. abortus* vaccine strain A19 was cultured, centrifuged at 5500 × *g* for 15 min, washed with PBS, and resuspended for colony-forming unit (CFU) counting on TSA plates. The cells were infected with *B. abortus* A19 at a multiplicity of infection (MOI) of 200. After 4 h, the cells were washed three times with PBS and cultured in DMEM/F-12 containing 50 µg/mL kanamycin for 1 h to eliminate extracellular bacteria. The medium was subsequently replaced with DMEM containing 10% FBS and 25 µg/mL kanamycin. At this point, AGK2 (10 µM, TargetMol, USA) was added to the cultures as needed.

### Flow cytometry analysis

The proportion of apoptotic cells was measured using flow cytometry (Keygen, KGA105, Nanjing, China). Briefly, BTCs in the logarithmic growth phase were seeded into 6-well plates at a density of 4 × 10^5^ cells per well and cultured for 12 h. The cells were infected with *B. abortus* A19 at an MOI of 200. After 48 h, the cells were centrifuged, washed twice with PBS, and incubated with 5 µL of FITC-labelled Annexin V and 5 µL of PI for 20 min at room temperature in the dark. Analysis was performed using a flow cytometer (BD FACSAria™ III, Franklin Lakes, NJ, USA), and the data were processed with FlowJo. At least 3 × 10^4^ cells were analysed for each measurement.

### Western blotting

BTCs in the logarithmic growth phase were seeded into 6-well plates at a density of 4 × 10^5^ cells per well and cultured for 12 h. The cells were infected with *B. abortus* A19 at an MOI of 200. After 48 h, the infected BTCs were centrifuged, washed with PBS, and lysed in an ice bath with lysis buffer (Keygen, KGP10100, Nanjing, China) for 30 min. The lysates were then centrifuged at 12 000 × *g* at 4 ℃ for 15 min, and the supernatants were collected. Protein concentrations were measured using a BCA kit (Keygen, KGP903, Nanjing, China). SDS loading buffer was added, and the samples were boiled at 100 ℃ for 10 min. The proteins were separated by 12% SDS‒PAGE and transferred onto PVDF membranes. The membranes were blocked with 10% nonfat milk for 2 h and incubated with the following primary antibodies for 24 h at 4 ℃: Bax (1:2000; Abmart, Shanghai, China), Caspase-3 (1:1000; Proteintech, Wuhan, China), SIRT2 (1:1000; Proteintech), P53 (1:2500; Proteintech), P53 (acetyl K370) (1:1000; MCE, America), Cyt-C (1:1000; Proteintech), DRP1 (1:1500; MCE), and β-actin (1:5000; Proteintech). After washing, the blots were incubated with an HRP-conjugated secondary antibody at room temperature for 1 h. The signal was detected with an enhanced chemiluminescence (ECL) chemiluminescence kit (Beyotime, P0018FS, Shanghai, China) and imaged with a gel imaging system (GE Amersham Imager 800, Boston, NY, USA).

### Assessment of changes in the mitochondrial membrane potential

BTCs in the logarithmic growth phase were seeded into 24-well plates at a density of 3 × 10^4^ cells per well. The cells were infected with *B. abortus* A19 at an MOI of 200. After 48 h of infection, the medium was aspirated, and the cells were washed twice with PBS, each lasting 5 min. JC-1 staining solution (Beyotime, Shanghai, China) was prepared according to the manufacturer’s instructions and added to the wells. The cells were then incubated at 37 ℃ with 5% CO_2_ for 30 min. After incubation, the cells were washed twice with 1 × buffer to remove excess staining solution, and 2 mL of fresh RPMI-1640 (HyClone, Logan, UT, USA) was added. Changes in the mitochondrial membrane potential were observed using a confocal laser scanning microscopy, and images were captured for analysis.

### Cellular ATP detection

Cellular ATP levels were measured using the ATP Assay Kit (Beyotime, Shanghai, China) following the manufacturer’s instructions. BTCs in the logarithmic growth phase were seeded into 6-well plates at a density of 4 × 10^5^ cells per well. The cells were infected with *B. abortus* A19 at an MOI of 200. After 48 h of *B. abortus* A19 infection, the culture medium was removed, and 200 μL of lysis buffer was added. The cells were lysed thoroughly by repeated pipetting. The lysates were subsequently centrifuged at 12 000 × *g* for 5 min at 4 ℃, after which the supernatants were collected for analysis. One hundred microliters of ATP detection solution was added to each sample and incubated at room temperature for 5 min to eliminate background ATP. An additional 20 μL of solution was then added to further reduce the background. RLU values were measured using a multifunctional enzyme label reader (BioTek, Winooski, VE, USA). The ATP concentration was calculated on the basis of a standard curve (0.01, 0.03, 0.1, 0.3, 1, 3, and 10 μM ATP) and converted to nmol/mg protein on the basis of the protein concentration.

### Measurement of reactive oxygen species formation

The levels of intracellular reactive oxygen species (ROS) were measured using a 2’,7’-dichlorofluorescein diacetate (DCFH-DA) probe (Beyotime, Shanghai, China), which is oxidation sensitive and non-fluorescent until it is oxidized to DCF. BTCs in the logarithmic growth phase were seeded into 24-well plates at a density of 3 × 10^4^ cells per well. After 48 h of *B. abortus* A19 infection at an MOI of 200, the cells were collected and incubated with DCFH-DA (diluted to 10 mM in serum-free medium at 1:1000) for 20 min at 37 ℃. The cells were then washed three times, observed for green fluorescence using a fluorescence inverted microscope, and photographed for analysis.

### RNA isolation and quantitative real-time PCR

Total cellular RNA was extracted using TRIzol (TaKaRa, Tokyo, Japan) and reverse-transcribed into cDNA using the Evo M-MLV RT reagent kit (AG Bio, Changsha, China). A 20 ng sample of cDNA was used as a template for analysing mRNA levels by quantitative real-time PCR (qPCR) with the SYBR qPCR Master Mix Kit (Vazyme Bio, Nanjing, China) on the Bio-Rad CFX96 system (Bio-Rad, Hercules, CA, USA) according to the manufacturer’s protocol. The primer sequences are listed in Additional file 1. The expression levels were evaluated using the 2^−ΔΔCt^ method. mRNA expression was normalized to that of the GAPDH gene, which served as the control for all samples.

### Extraction of nuclear and cytoplasmic proteins

Nuclear and cytoplasmic proteins were extracted using the Nuclear and Cytoplasmic Protein Extraction Kit (WANLEIBIO, Shenyang, China) according to the manufacturer’s instructions. BTCs in the logarithmic growth phase were seeded into 6-well plates at a density of 4 × 10^5^ cells per well. The cells were infected with *B. abortus* A19 at an MOI of 200. After 48 h, the cell samples were collected. An appropriate amount of Cytoplasmic Protein Reagent A containing PMSF was added, mixed thoroughly, and incubated on ice for 15 min. Next, Cytoplasmic Protein Reagent B was added, mixed well, and incubated on ice for 1 min, followed by centrifugation. The supernatant containing the cytoplasmic proteins was collected and transferred to an EP tube. The remaining pellet was resuspended in 50 µL of Nuclear Protein Reagent, mixed thoroughly, and incubated on ice for 30 min with vortexing every 2 min. The mixture was then centrifuged at 12 000 rpm for 10 min at 4 ℃. The supernatant, containing the nuclear proteins, was collected.

### Immunofluorescence

BTCs were seeded on glass coverslips in 24-well plates at a density of 3 × 10^4^ cells per well. The cells were infected with *B. abortus* A19 at an MOI of 200. After 48 h of incubation with *B. abortus* A19, the cells were fixed with 4% paraformaldehyde and permeabilized with 0.2% Triton X-100 in PBS for 30 min at room temperature. The slides were then washed three times with PBS. For immunostaining, the slides were incubated overnight at 4 ℃ with primary antibodies against caspase-3 (1:500; Wuhan, China; Proteintech) and SIRT2 (1:500; Proteintech). The following day, the slides were stained for 2 h at room temperature with Alexa-Fluor 488-conjugated donkey anti-rabbit IgG (for SIRT2, 1:500, A32790, Thermo Fisher). Nuclei were stained with DAPI for 15 min. After each incubation step, the slides were washed four times with PBS. Images were captured using a confocal microscope (A1R; Nikon, Tokyo, Japan).

### CFU counting of infected BTCs

BTCs were infected with *B. abortus* A19 at an MOI of 200. At the indicated time points (0, 6, 12, 24, and 48 h), the cells were washed three times with PBS and lysed with PBS containing 0.5% Triton X-100 for 10 min. The lysates were serially diluted with PBS, plated on TSA, and incubated at 37 ℃ for 72 h.

### Statistical analysis

All experiments were independently repeated at least three times, and the data are expressed as the mean ± standard deviation (SD) of three replicate experiments. Statistical analysis was performed using SPSS software (SPSS, Inc., Cary, NC, USA). Differences between groups were analysed via one-way analysis of variance (ANOVA) followed by Tukey’s post hoc test. A *p* value of < 0.05 was considered to indicate statistical significance.

## Results

### *Brucella* infection induces apoptosis in BTCs

**Figure 1:**
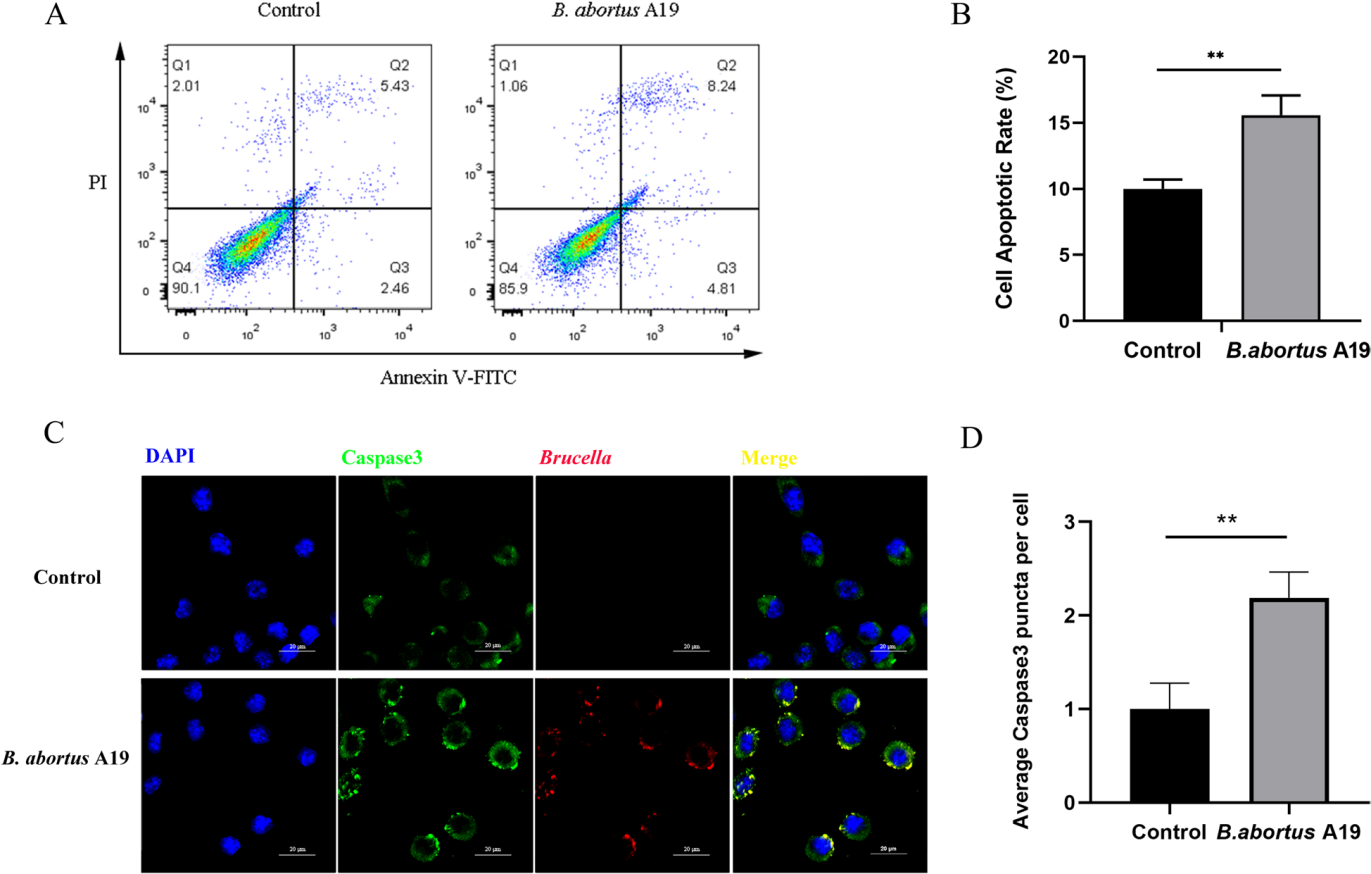
***B. abortus* A19 induces apoptosis in BTCs**. **A** Flow cytometry analysis of BTCs collected at 48 h post-infection with *B. abortus* A19 and stained with FITC-labelled Annexin V and propidium iodide. **B** Statistical analysis of the percentage of apoptotic BTCs. **C** Protein expression of Caspase3. Bars: 20 µm. **D** The average number of Caspase3 puncta per cell in BTCs. The experiment was repeated three times. The data represent mean ± SD. ** *p* < 0.01, as analysed with one-way ANOVA and the Bonferroni multiple comparison test.

The modulation of host cell apoptosis is a typical survival strategy used by infectious pathogens. The impact of *B. abortus* A19 infection on cell apoptosis in BTCs was examined using flow cytometry with Annexin V and propidium iodide staining. The percentage of apoptotic cells after 48 h was 15.23% for the infected group, whereas it was 10.57% for the control group (Figures 1A and B). Subsequent immunofluorescence revealed that infection with *B. abortus* A19 for 48 h resulted in increased expression of total caspase-3 (Figures 1C and D). These results indicate that *B. abortus* A19 induces apoptosis in BTCs after 48 h of infection.

### *Brucella* infection induces mitochondrial damage in BTCs

**Figure 2:**
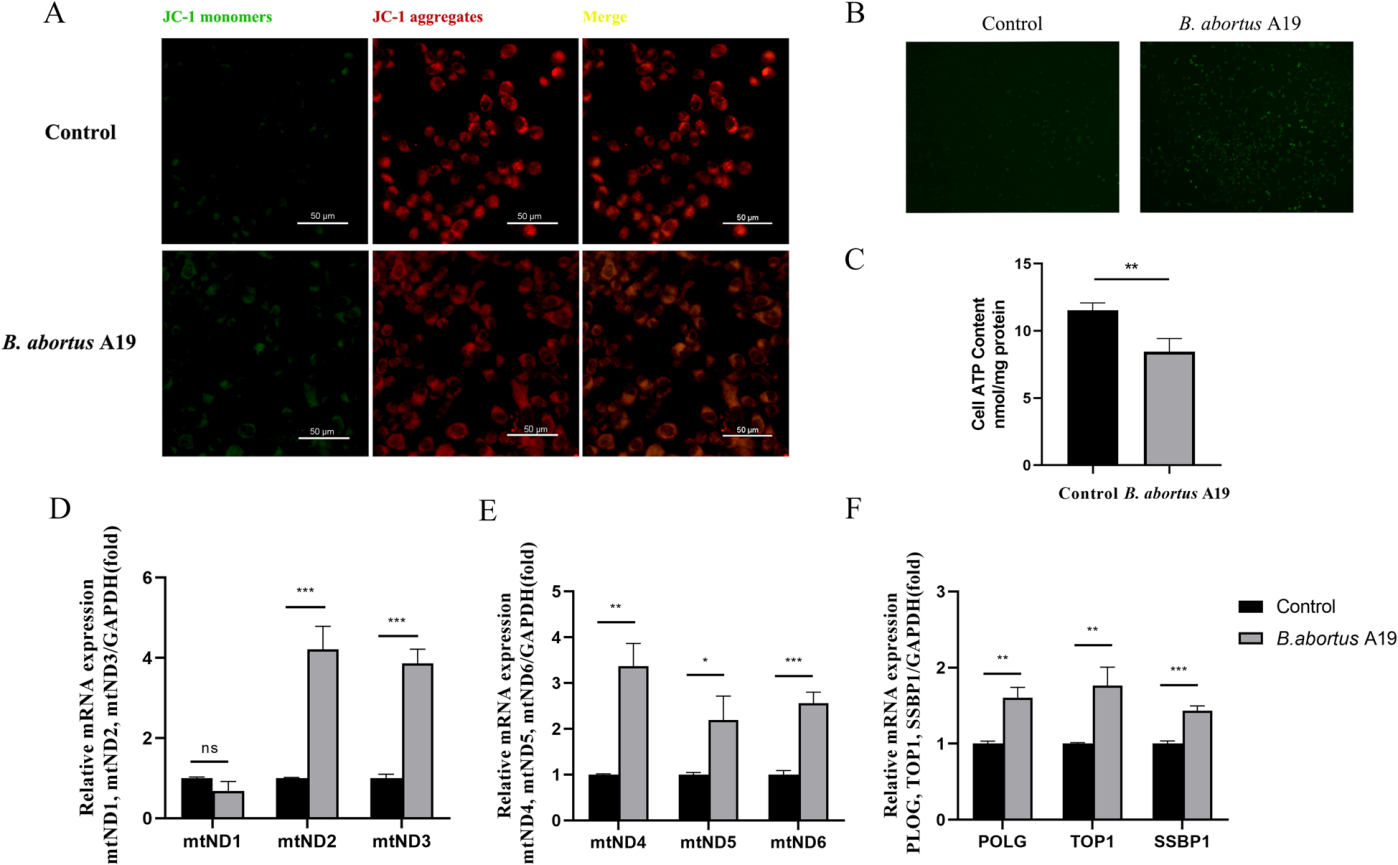
**Mitochondrial damage caused by *B. abortus* A19 infection in BTCs**. **A** JC-1 staining for visualizing the mitochondrial membrane potential. Scale bar: 50 µm. **B** Detection of ROS levels. **C** Detection of ATP levels. **D** The mRNA levels of mitochondrial DNA1-3. **E** The mRNA levels of mitochondrial DNA4-6. **F** mRNA levels of POLG, TOP1, and SSBP1. The experiment was repeated three times. The data represent mean ± SD. *p < 0.05; **p < 0.01; ***p < 0.001, as analysed with one-way ANOVA and the Bonferroni multiple comparison test.

Mitochondria are the sites of energy synthesis and storage, as well as material metabolism and energy conversion in cells. As executors of apoptosis, mitochondria induce cell apoptosis when damaged. Therefore, we investigated whether *B. abortus* A19 affects mitochondrial damage in BTCs after 48 h of infection. JC-1 staining was used to evaluate the extent of mitochondrial damage, and we observed a significant reduction in the mitochondrial membrane potential in the *B. abortus* A19 infection group (Figure 2A). Additionally, when the fluorescence probe DCFH-DA was used to detect the ROS levels, we found that the ROS levels in the BTCs of the infection group were significantly greater than those in the control group (Figure 2B), whereas the ATP levels were significantly lower (Figure 2C). These results indicate that mitochondrial function in BTCs is impaired by *B. abortus* A19 infection. Furthermore, we used RT‒qPCR to detect changes in the expression of mitochondrial DNA (mtDNA1-6) and nuclear genome genes that control mitochondrial DNA replication (POLG, SSBP1, and TOP1). The results revealed that 48 h after infection with *B. abortus* A19, the mRNA expression of mtDNA2-6, POLG, SSBP1, and TOP1 in the BTC group was significantly greater than that in the control group (Figures 2D–F). These findings suggest that *B. abortus* A19 infection induces mitochondrial damage in BTCs.

### *Brucella* infection upregulates the expression of SIRT2 in BTCs

**Figure 3:**
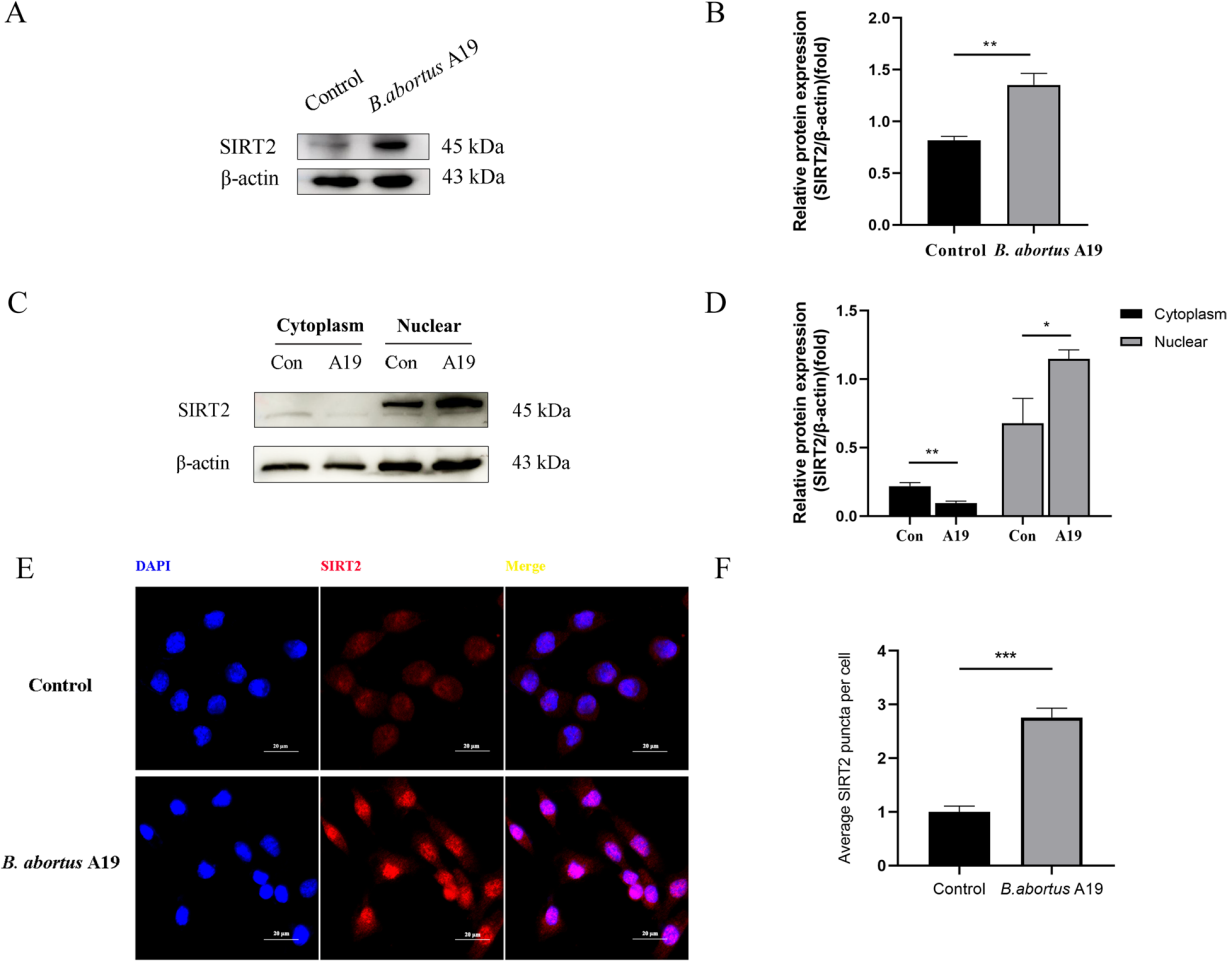
**Upregulation of SIRT2 by *B. abortus* A19 infection in BTCs**. **A** Protein expression of SIRT2. **B** The ratio of the SIRT2 level to the β-actin level. **C** Expression of SIRT2 in the cytoplasm and nucleus is regulated by *B. abortus* A19 infection. **D** The ratio of SIRT2 in the cytoplasm and nucleus relative to the β-actin level. **E** Protein expression of SIRT2; scale bars: 20 µm. **F** Average number of SIRT2 puncta per cell. The experiment was repeated three times. The data represent mean ± SD. **p < 0.01; ***p < 0.001, as analysed with one-way ANOVA and the Bonferroni multiple comparison test.

To investigate the potential role of SIRT2 in BTCs infected with *B. abortus* A19, we examined the changes in the expression of SIRT2 after 48 h of infection. We found that SIRT2 protein expression in BTCs was upregulated by *B. abortus* A19 infection at 48 h (Figures 3A and B). SIRT2 is the only major Sirtuin that is primarily localized in the cytoplasm; however, during mitosis and pathogen infection, SIRT2 shuttles the nucleus and regulates chromatin condensation [17]. To explore whether this phenomenon occurs during *B. abortus* A19 infection in BTCs, we analysed the changes in the expression of SIRT2 in the cytoplasm and nucleus by western blotting. The results indicated that SIRT2 protein expression was downregulated in the cytoplasm and upregulated in the nucleus by *B. abortus* A19 infection at 48 h (Figures 3C and D). To further confirm the protein expression levels of SIRT2, we used immunofluorescence to detect the expression of SIRT2 in BTCs at 48 h post-infection. The results revealed that SIRT2 expression was significantly greater in the infected group than in the control group (Figures 3E and F), which was consistent with the protein expression levels.

### SIRT2 inhibits *Brucella*-induced mitochondrial apoptosis in BTCs

**Figure 4:**
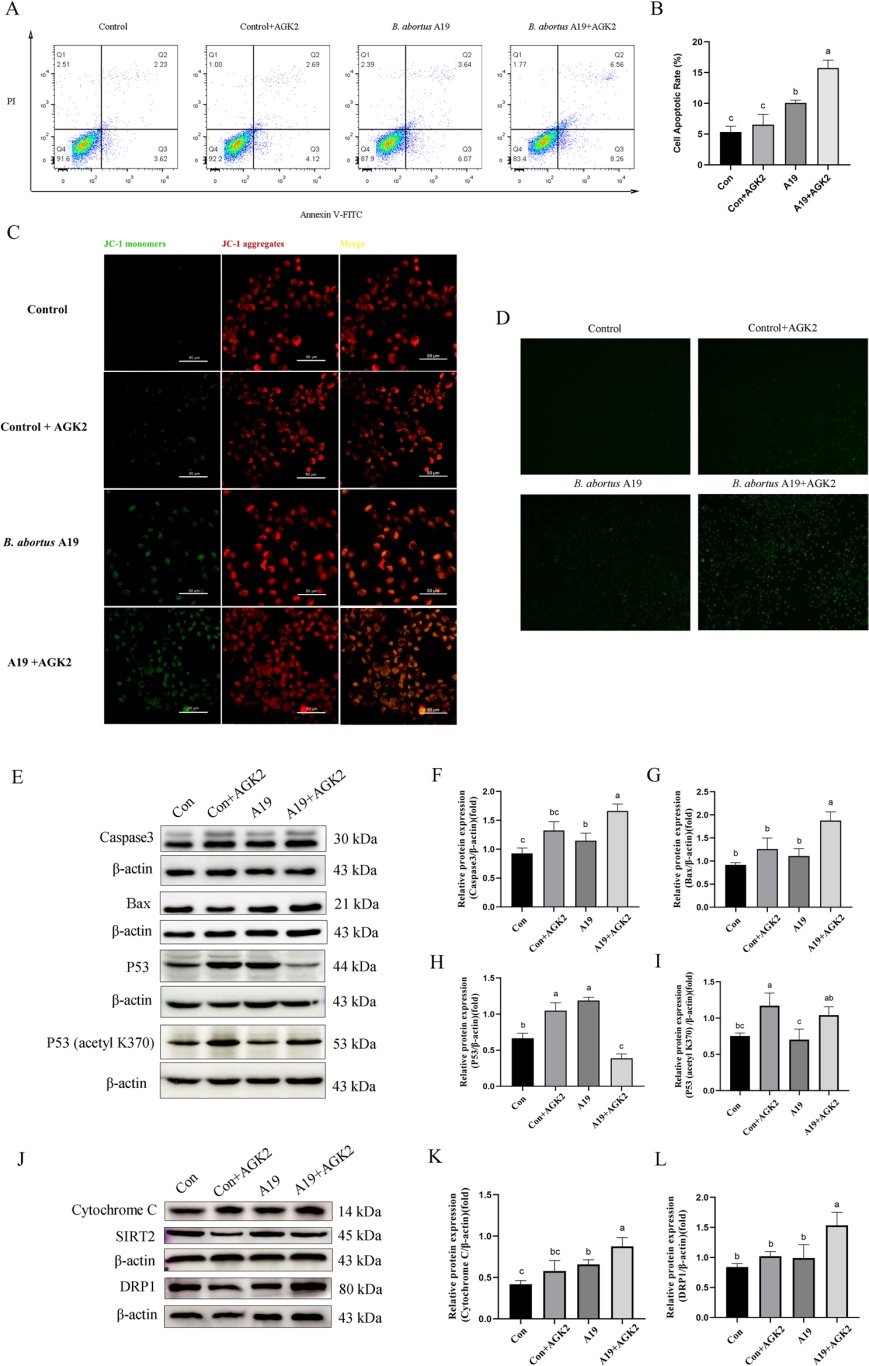
**SIRT2 suppresses *Brucella*-induced mitochondrial apoptosis**.**A** Effect of SIRT2 inhibition on *B. abortus* A19-induced cell apoptosis, as determined by flow cytometry. **B** Percentage of apoptotic cells. **C** Changes in the mitochondrial membrane potential determined by JC-1 staining. Scale bar: 50 µm. **D** DCFH-DA staining was used to measure the ROS levels. **E** Expression of Caspase3, Bax, P53 and P53 (acetyl K370). **F** The ratio of Caspase3 relative to β-actin. **G** The ratio of Bax relative to β-actin. **H** The ratio of P53 relative to β-actin. **I** The ratio of P53 (acetyl K370) relative to β-actin. **J** Expression of Cyt-C and DRP1. **K** The ratio of Cyt-C to β-actin. **L** The ratio of DRP1 to β-actin. The experiment was repeated three times. The data represent the mean ± SD. Bars with different letters indicate significant differences (*p* < 0.05).

To assess the role of SIRT2 in regulating *Brucella*-induced apoptosis, BTCs were infected with *B. abortus* A19, followed by the addition of 10 µM AGK2, a SIRT2 inhibitor, to fresh culture medium. The optimal concentration of AGK2 was determined by measuring cell viability using CCK8 (Additional file 2). We employed flow cytometry to evaluate the impact of SIRT2 inhibition on apoptosis in BTCs. The results showed that SIRT2 inhibition significantly increased the apoptosis rate in the infected group (Figures 4A and B), which suggested that SIRT2 inhibits *Brucella*-induced apoptosis.

A small amount of SIRT2 is localized in mitochondria and participates in regulating mitochondrial function [28]. Therefore, we hypothesized that SIRT2 influences *Brucella*-induced apoptosis by regulating the mitochondrial apoptosis pathway. To verify this hypothesis, we measured changes in the mitochondrial membrane potential and ROS levels. The results revealed that the inhibition of SIRT2 by *B. abortus* A19 infection led to a decrease in the mitochondrial membrane potential (Figure 4C) and an increase in the ROS level (Figure 4D). We then used Western blotting to examine the effects of SIRT2 inhibition on the expression of Caspase-3, Bax, P53, acetylated P53 (K370), Cyt-C, and DRP1 in BTCs. We observed that SIRT2 inhibition increased Caspase-3 and Bax expression (Figures 4E-G), which induced apoptosis. It also decreased P53 protein levels (Figures 4E and H) and increased acetylated P53 (K370) levels (Figures 4E and I), indicating that SIRT2 may regulate apoptosis by deacetylating P53 (K370). Additionally, Cyt-C expression increased (Figures 4J and K), affecting the mitochondrial electron transport chain, and DRP1 expression increased (Figures 4J and L), implying disrupted mitochondrial fission in BTCs infected with *B. abortus* A19.

### SIRT2 promotes *Brucella* replication

**Figure 5:**
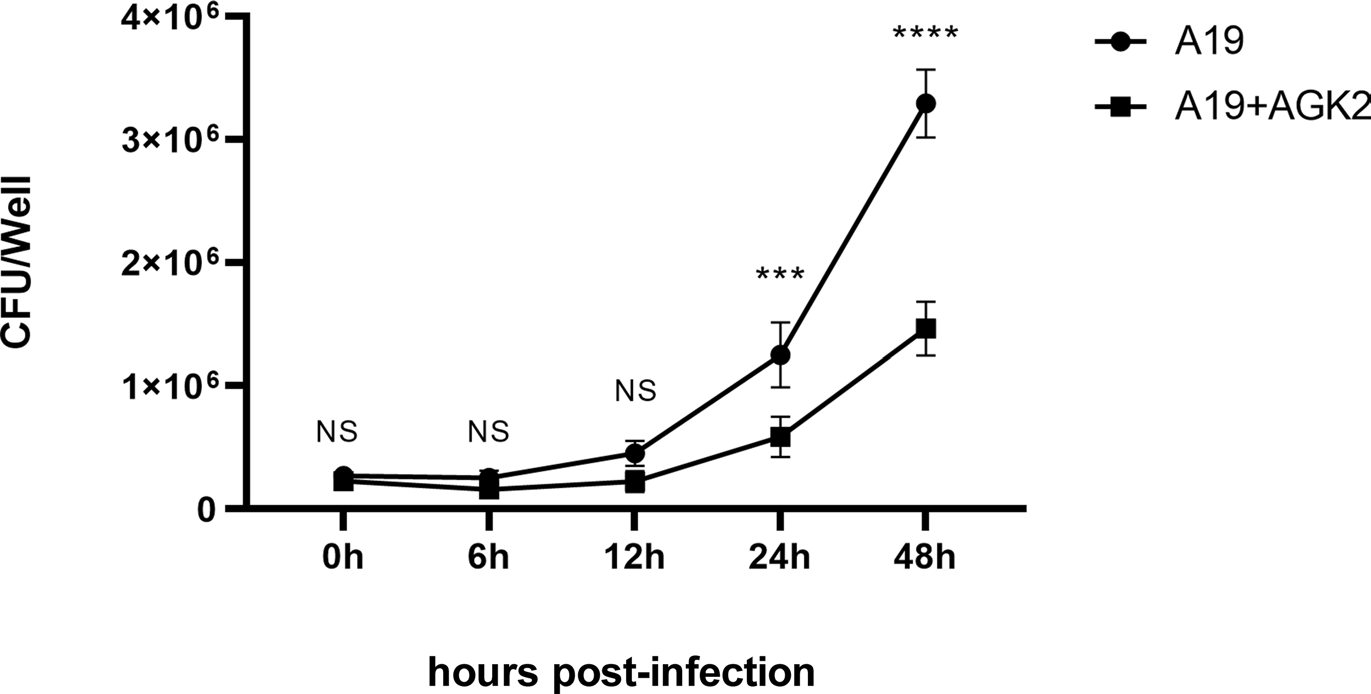
**Inhibition of SIRT2 reduces* B. abortus* A19 replication**. The experiment was repeated three times. The data represent the mean ± SD. *** *p* < 0.001 by one-way ANOVA and post hoc Bonferroni multiple comparison test.

To determine the effect of SIRT2 on *Brucella* replication, we infected BTCs with *B. abortus* A19 and then added 10 µM of the SIRT2 inhibitor AGK2. Bacterial colony counts were measured at 0 h, 6 h, 12 h, 24 h, and 48 h. The results revealed that there were no significant differences in colony counts at 0 h, 6 h, and 12 h. However, at 24 h and 48 h, the inhibition of SIRT2 significantly reduced the number of intracellular *Brucella* in BTCs (Figure 5). These findings indicate that SIRT2 promotes *Brucella* replication.

## Discussion

The primary pathogenic mechanism of *Brucella* involves complex interactions with host cells, where regulating host cell apoptosis is a necessary step for *Brucella* to achieve its intracellular lifecycle [29, 30]. *Brucella* can either promote or delay apoptosis under different conditions. Caspase3, one of the most crucial executors of the apoptosis process, plays a major role in apoptosis. Research has indicated that *B. abortus* infection in macrophages activates Nedd4, leading to the degradation of Calpain2, which in turn inhibits the activation of Caspase3, thus suppressing apoptosis [31]. ROS serve as second messengers in apoptosis [32]. When cells receive apoptotic signals, ROS levels increase, which may lead to increased Ca^2+^ influx, upregulation of Bax, opening of the mitochondrial permeability transition pore (MPTP), activation of trypsin, and ultimately, cell death [33]. Different levels of ROS determine whether cells undergo apoptosis, necrosis, or the transition from apoptosis to necrosis. *B. melitensis* 16 M can regulate the effect of the AIR domain on apoptosis in mouse macrophages through the ROS signalling pathway. As the infection time increased, the ability of *B. melitensis* 16 M to promote apoptosis increased. AIR can also affect *B. melitensis* 16 M-induced apoptosis via the ROS pathway [34]. In this study, we observed through flow cytometry that *B. abortus* A19 infection induced apoptosis in BTCs after 48 h. Further immunofluorescence results revealed a significant increase in total caspase-3 expression following 48 h of infection. These findings indicate that *B. abortus* A19 induces apoptosis in BTCs, but its cellular targets and specific mechanisms remain unclear.

Mitochondria play a critical role in the apoptotic process. When the intrinsic apoptotic pathway, characterized by the opening of the mitochondrial permeability transition pore and the loss of mitochondrial membrane potential, is activated, cytochrome C (Cyt-C) is released from mitochondria via Bax and Bak from the Bcl-2-like family. In the cytoplasm, Cyt-C activates caspases, leading to programmed cell death [35, 36]. Typically, when a host is infected by pathogenic microorganisms, mitochondria can induce an immune response to eliminate infected cells. Consequently, some pathogens may evolve various escape mechanisms to avoid being cleared by the host, whereas mitochondria may evolve corresponding mechanisms to resist pathogen infection. Studies have shown that *Pseudomonas aeruginosa* can induce mitochondrial damage, leading to lung injury and multiple organ failure [37]. *Salmonella* Typhimurium has also been found to cause cytosolic leakage of mtDNA and promote cGAS-STING signalling [38]. Both *Mycobacterium abscessus* and *Mycobacterium tuberculosis* can induce mitochondrial damage and mtDNA-mediated inflammatory signalling through the inflammasome or cGAS-STING pathways [39, 40]. In this study, *B. abortus* A19 infection in BTCs caused mitochondrial dysfunction, characterized by decreased membrane potential, increased ROS levels, reduced ATP levels, and elevated mtDNA expression after 48 h. The expression of nuclear genes that regulate mitochondrial replication was also correlated with mtDNA expression.

Histone acetyltransferases (HATs) and histone deacetylases (HDACs) play crucial roles in chromatin structure modification and gene expression regulation. Previous studies have reported that microbial infections can affect the acetylation levels of host proteins [41]. As a key deacetylase in host cells, SIRT2 plays an important role in regulating various biological processes, including cell apoptosis, cell proliferation, oxidative stress, and host‒pathogen interactions [42]. In this study, AGK2, a commonly used selective and specific SIRT2 inhibitor, was used to inhibit the deacetylase activity of SIRT2. This inhibitor has been applied in various cell models and has different effects, such as affecting cell cycle arrest, reducing cellular ATP levels, and inducing late apoptosis and necrosis [43]. As a well-established SIRT2 inhibitor, some studies using AGK2 at a concentration of 10 µM in mouse models have reported no significant side effects [44]. In various bacterial infection models, such as *Mycobacterium tuberculosis*, *Salmonella*, and *Listeria monocytogenes*, AGK2 chemical inhibitors have been used to inhibit SIRT2 biological activity to explore the role of SIRT2 in bacterial pathogenic mechanisms. This study screened a drug concentration of 10 µM for BTCs. At this concentration, the expression of the SIRT2 protein in BTCs was significantly inhibited after 48 h, and this concentration did not affect the viability of *Brucella* itself. Therefore, 10 µM was chosen as the working concentration of AGK2.

SIRT2 plays a role in the apoptosis of various cell types. Studies have shown that downregulation of SIRT2 can induce apoptosis in cancer cells and mediate oxidative stress-induced apoptosis [45, 46]. These findings indicate that SIRT2 has an antiapoptotic function. However, the role of SIRT2 in apoptosis may be cell type specific. Research has shown that SIRT2 does not participate in stress-induced apoptosis in the human epithelial cancer cell line HCT116 [47]. Moreover, the SIRT family protein SIRT1 is also a crucial antiapoptotic molecule in certain leukemia cells, indicating potential interactions between different SIRT proteins in regulating apoptosis [48]. Recent studies have shown that a small amount of SIRT2 is localized in mitochondria and is involved in regulating their function [49]. In bovine oocytes during meiosis, the use of a SIRT2 inhibitor led to mitochondrial dysfunction, increased ROS levels, and upregulation of the mitochondrial fission marker DRP1. This imbalance causes mitochondrial dynamics abnormalities in bovine oocytes during meiosis [50]. This study investigated the role of SIRT2 in *Brucella*-induced mitochondrial apoptosis in BTCs. After 48 h of *Brucella* infection and AGK2 treatment, the results revealed that AGK2 increased *Brucella*-induced apoptosis and reduced the bacterial load. In the AGK2 group, the mitochondrial membrane potential was lower, and the ROS levels were greater, indicating impaired mitochondrial function due to SIRT2 inhibition. Additionally, SIRT2 inhibition significantly decreased the expression of the mitochondrial fission protein DRP1 in infected cells.

There is a complex interaction between SIRT2 and P53. Studies have shown that SIRT2 can interact with P53 and decrease its activity [51]. Downregulation of SIRT2 can lead to P53 accumulation and apoptosis in cancer cells [45]. Therefore, we speculate that in the process of *Brucella*-induced apoptosis in BTCs, SIRT2 may participate in regulating P53 expression to inhibit apoptosis. Our results revealed that in the infected group, SIRT2 inhibition for 48 h significantly reduced total P53 protein expression, and acetylated P53 (K370) levels significantly increased, indicating that SIRT2 may inhibit *Brucella*-induced BTC apoptosis by deacetylating P53 (K370). However, the specific molecular mechanisms by which SIRT2 regulates P53 require further investigation.

In summary, this study investigated the impact of SIRT2 on the intracellular survival of *Brucella* and *Brucella*-induced mitochondrial apoptosis in trophoblast cells, revealing the role of SIRT2 in the pathogenic mechanism of *Brucella*. These findings provide a theoretical basis for exploring effective host-directed therapies for brucellosis, offering new insights into how *Brucella* evades immune clearance through host cell regulatory mechanisms. However, this study faces challenges and unresolved questions. First, given that SIRT2, along with other sirtuins, are multifunctional enzymes with complex roles, their inhibition may lead to diverse and potentially unpredictable effects. Therefore, understanding these intricate mechanisms is crucial before considering SIRT2 as a therapeutic target. Second, other signalling pathways and regulatory mechanisms induced by *Brucella* infection remain unclear; future research should focus on these aspects to comprehensively understand the complex network of interactions between *Brucella* and host cells. In conclusion, this study not only reveals the role of SIRT2 in regulating cell apoptosis during *Brucella* infection but also provides new directions for the development of targeted therapies against brucellosis.

## Supplementary Information

**Additional file 1. The sequences of primers used for RT‒qPCR.**

**Additional file 2. Cell viability was measured by a CCK8 assay**. The experiment was repeated three times. The data represent the mean ± SD. * *p* < 0.05; ** p < 0.01, as analysed with one-way ANOVA and the Bonferroni multiple comparison test.
