# Supplementary material for: SIRT2 inhibition enhances mitochondrial apoptosis in Brucella-infected bovine placental trophoblast cells
Source: Vet Res. 2025 May 2;56:97. doi: 10.1186/s13567-025-01518-8 (PMC12049057; doi:10.1186/s13567-025-01518-8)
Supplement: Supplementary file 1 — Additional file 1. The sequences of primers used for RT‒qPCR. [file 13567_2025_1518_MOESM1_ESM.docx]

**Additional file 1 The primer sequences of RT-qPCR.**

| Gene | Forward Primer (5’- 3’) | Reverse Primer (5’- 3’) |
| --- | --- | --- |
| mtDNA1 | CTAGTCTCGGGCTTCAACG | GGTAGGATGCTCGGATT |
| mtDNA2 | AGCCTACTCATCAATCGC | TGACAGGGTAGTGGTGGT |
| mtDNA3 | CATCGCATTCTGACTTCC | ATTTGCTGTTTGTGAGGC |
| mtDNA4 | TTACCCGATGAGGAAACC | ATAACGAGTGCTATGTGGC |
| mtDNA5 | TGATACGGACGAGCAGAT | TTGAGTCGCTTGGGTTTA |
| mtDNA6 | CTGTAGCCATAGCCGTTGT | GGGTTAGGGTTAATTGTGAGT |
| POLG | AAGGCGAAGAAAGGGAAGA | TGGAGGCTGAGGAGACTGG |
| SSBP1 | AGGCATGAGTCTGAAGTAGCT | CGGTCGGAACACTGAAAT |
| TOP1 | AGTCCGACACGATAACAAG | CGATGAAGTACAGGGCTAC |
