# Supplementary material for: SIRT2 inhibition enhances mitochondrial apoptosis in Brucella-infected bovine placental trophoblast cells
Source: Vet Res. 2025 May 2;56:97. doi: 10.1186/s13567-025-01518-8 (PMC12049057; doi:10.1186/s13567-025-01518-8)
Supplement: Supplementary file 2 — Additional file 2. Cell viability was measured by a CCK8 assay. The experiment was repeated three times. The data represent the mean ± SD. * p < 0.05; ** p < 0.01, as analysed with one-way ANOVA and the Bonferroni multiple comparison test. [file 13567_2025_1518_MOESM2_ESM.docx]

**Additional file 2 The cell viability was measured by CCK8.**


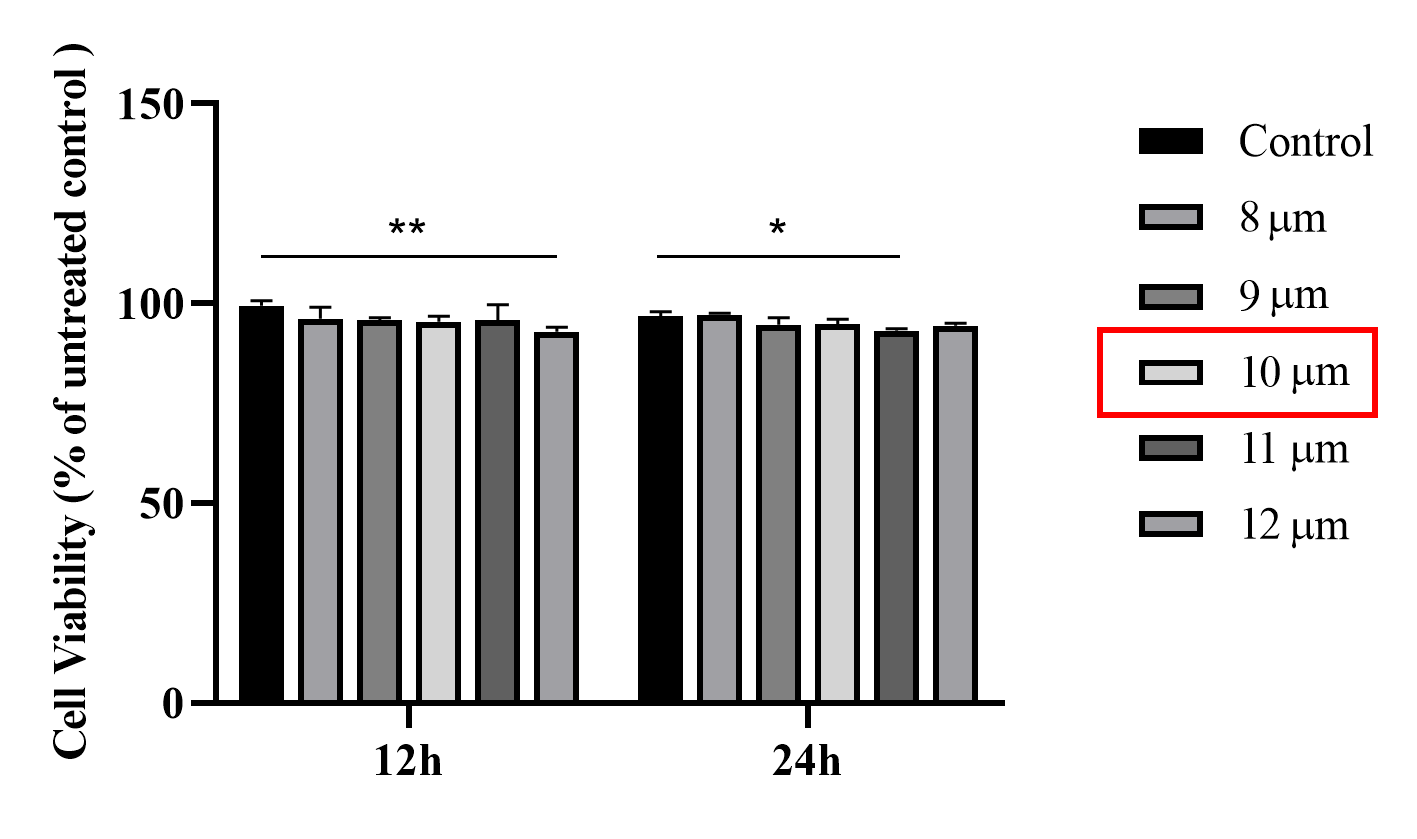


The experiment was repeated three times. Data represent mean ± SD. * *p* < 0.05; ** *p* < 0.01, as analyzed with one-way ANOVA and Bonferroni multiple comparison test.
